# Dynamic stability of climate-growth relationships in *Picea crassifolia* in the Qilian Mountains: the modulating role of elevation gradient and time-varying characteristics

**DOI:** 10.3389/fpls.2026.1782742

**Published:** 2026-03-16

**Authors:** Jingzhong Zhao, Yuan Gao, Xiurong Wu, Xiaofeng Ren, Xuee Ma, Hao Yuan, Weijun Zhao, Nan Zhao, Michael Vrahnakis, Aristeidis Kastridis, Na Wei, Wenmao Jing

**Affiliations:** 1 Gansu Qilian Mountain Water Conservation Forest Research Institute, Zhangye, China; 2 Gansu Qilian Mountain Forest Ecosystem Research Station, Zhangye, China; 3 Management Center of Gansu Qilian Mountain National Nature Reserve, Zhangye, China; 4 Department of Forestry, Wood Sciences and Design, University of Thessaly, Karditsa, Greece

**Keywords:** dendroclimatology, elevation gradient, *Picea crassifolia*, Qilian Mountains, tree-ring width chronologies

## Abstract

**Introduction:**

Amid ongoing climate warming, *Picea crassifolia* in the arid and semi-arid Qilian Mountains has exhibited increasingly unstable growth responses to climatic variability, raising concerns regarding the resilience of high-elevation forests in this ecologically sensitive region. To elucidate the modulating effect of elevation gradients on the stability of tree-climate relationships, this study examined *Picea crassifolia*, the dominant conifer species in the region.

**Methods:**

Tree-ring width chronologies were developed from samples collected across five elevation bands (2,900-3,300 m a.s.l.) within the Pailugou watershed of the Qilian Mountains. By integrating Climate Research Unit (CRU) gridded climate data and applying 30-year moving window correlation analyses, this study systematically evaluated the temporal stability of climate-growth relationships along the elevation gradient.

**Results:**

The results are as follows: (1) At lower elevations (2,900-3,000 m a.s.l.), tree growth was primarily limited by moisture availability and influenced by summer temperatures, exhibiting significant positive correlations (p < 0.01) with precipitation in January and September of the current year. In contrast, at higher elevations (3,200-3,300 m a.s.l.), trees exhibited greater sensitivity to winter conditions, demonstrating significant negative correlations (p < 0.05) with December precipitation of the previous year and June temperature of the current year. (2) Moving window correlation analyses between tree-ring chronologies and climate variables revealed nonstationary climate-growth relationships across all five elevation sites. Notably, trees at mid- to high-elevation sites (3,100-3,300 m a.s.l.) exhibited pronounced temporal variability in their climate-growth responses, particularly during the growing season (June-September) and adjacent months. This instability is likely attributable to intensifying hydrothermal imbalances driven by climate warming.

**Discussion:**

This study demonstrates that elevation gradients modulate the temporal stability of climate–growth relationships and underscores that the responses of alpine forest ecosystems to climate change are dynamic rather than static. These findings provide a novel framework for understanding the dynamic adaptive mechanisms of montane ecosystems in the Qilian Mountains under ongoing climate change and offer critical insights for designing adaptive forest management strategies in high-elevation cold regions.

## 1 Introduction

Global climate change is profoundly altering the structure and function of mountain ecosystems, positioning alpine ecosystems a focal point of ecological research due to their high sensitivity to climatic shifts (IPCC, 2023). As a critical component of Earth’s terrestrial ecosystems, forests not only provide essential ecosystem services to human societies but also play a pivotal role in maintaining ecological balance (Alkama and Cescatti, 2016). Climate change profoundly influences the trajectory of forest ecosystem development, and concurrent changes in forest dynamics serve as sensitive indicators of broader climatic trends (Körner and Basler, 2010; Lu et al., 2019).

The response of forest ecosystems to global climate change exhibits pronounced spatial heterogeneity, particularly along elevational gradients (Keenan, 2015). Numerous studies have demonstrated that tree radial growth responds to interannual climatic variability, yet these relationships are often non-stationary over time (Zhang et al., 2009). Growth-climate relationships exhibit instability for *Pinus koraiensis* in Changbai Mountains, *Picea schrenkiana* in the Alatau Mountains, and *Abies faxoniana* in western Sichuan (Jiao et al., 2015; Zhang et al., 2019; Altman et al., 2020). Conversely, growth responses to limiting climatic factors remain relatively stable for *Larix decidua* in the European Alps, *Picea schrenkiana* in the western Tianshan Mountains, and *Picea crassifolia* in northwestern China (Büntgen et al., 2008; Zhang et al., 2020).The main influencing factors include drought stress caused by warming and drying, temperature thresholds, changes in atmospheric CO_2_ concentration, tree age, and micro-environmental conditions (Lamarche et al., 1984; Yu et al., 2006; Gai et al., 2017). Under climate warming, intensified soil moisture loss through evaporation reduces soil water availability, heightening tree sensitivity to moisture conditions (Zhang et al., 2009). Consequently, radial growth is increasingly co-limited by temperature and precipitation, supplanting the historical paradigm of single-factor (temperature) limitation.

In montane environments, elevation serves as a primary determinant of tree growth performance and constitutes a critical proxy for evaluating climate change impacts on radial growth (Malanson et al., 2011; Wang et al., 2015). Climatic conditions-including temperature, precipitation, solar radiation, and associated topographic influences-vary markedly across elevation zones. A growing body of evidence confirms that climatic drivers exert differential effects on the radial growth of a given tree species across elevational gradients, underscoring the climate-dependence of tree growth. At high elevations, low temperatures typically constrain radial growth, whereas at lower elevations, water availability-primarily governed by precipitation-represents the principal limiting factor (Chen et al., 2014). Specifically, radial growth at high elevations is predominantly limited by thermal constraints, whereas at mid- to low elevations, it is increasingly constrained by drought stress resulting from elevated temperatures and reduced moisture availability (White et al., 2014). For instance, research in the northern Greater Khingan Range demonstrated that radial growth of *Larix gmelinii*-a dominant boreal conifer-is co-limited by temperature and precipitation (Qin et al., 2024). However, this paradigm is not universally applicable, as regional differences in climate and environmental context modulate species-specific growth responses (Shi et al., 2019; Wang et al., 2005). For instance, in the arid and semi-arid mountains of northwestern Argentina and Central Asia, radial growth at high elevations is primarily driven by precipitation rather than thermal limitations (Morales et al., 2004; Liu et al., 2006). Under ongoing global warming, precipitation has increasingly emerged as a key limiting factor for tree growth even at high elevations in arid and semi-arid mountain systems. Consequently, the mechanisms governing tree responses to climate change across elevational gradients remain incompletely understood, warranting in-depth investigation into climate-growth dynamics.

The Qilian Mountains, situated at the northeastern margin of the Tibetan Plateau, constitute a representative cold-arid to semi-arid region in China, characterized by a continental climate with pronounced moisture deficits. Moreover, this region lies at the intersection of the East Asian monsoon and mid-latitude westerlies, rendering its climate highly sensitive to global climatic perturbations (Chen et al., 2008; Fang et al., 2010). In recent decades, the Qilian Mountains region has exhibited a pronounced warming-wetting trend; however, this trend displays significant spatial heterogeneity, with a greater magnitude of temperature increase observed in the eastern section and a greater magnitude of precipitation increase in the central and western sections (Yin et al., 2009). *Picea crassifolia* is the dominant conifer in the Qilian Mountains and a keystone species in arid and semi-arid montane forests, exhibiting strong drought resistance, cold tolerance, and high climate sensitivity (He et al., 2016). Distributed in belt-like or patchy patterns on the north-facing and semi-shaded slopes between 2,700 and 3,300 m elevation, *Picea crassifolia* forests occupy 75.72% of the total forest area in the region (Tian et al., 2012). Influenced by regional climatic conditions and anthropogenic activities, tree growth of *Picea crassifolia* exhibits spatial inconsistency between the eastern and central regions. Therefore, there is an urgent need to understand the differential responses of these forests to climatic factors under varying regional climate change scenarios.

Dendrochronology, with its annually resolved chronologies, precise dating, high temporal fidelity, and capacity for replication, has become a cornerstone method for reconstructing past climate variability and deciphering climate-growth relationships (Yu et al., 2016). Accordingly, investigating the climatic drivers of radial growth in *Picea crassifolia* across the central Qilian Mountains is of considerable scientific relevance. This study focuses on *Picea crassifolia* in the Pailugou watershed of the central Qilian Mountains, where standardized tree-ring width chronologies were developed across five elevation bands (2,900-3,300 m a.s.l.). Climate-growth relationships were analyzed across these five elevation bands with three objectives: (1) to identify the primary climatic constraints at each elevation; (2) to characterize elevational patterns in the temporal stability of growth responses; and (3) to evaluate how the influence of specific climatic variables on radial growth varies with elevation. These findings advance understanding of forest-climate feedbacks in high-elevation arid ecosystems and provide a scientific basis for adaptive forest management and sustainable conservation strategies in the Qilian Mountains.

## 2 Material and methods

### 2.1 Study area

**Figure 1:**
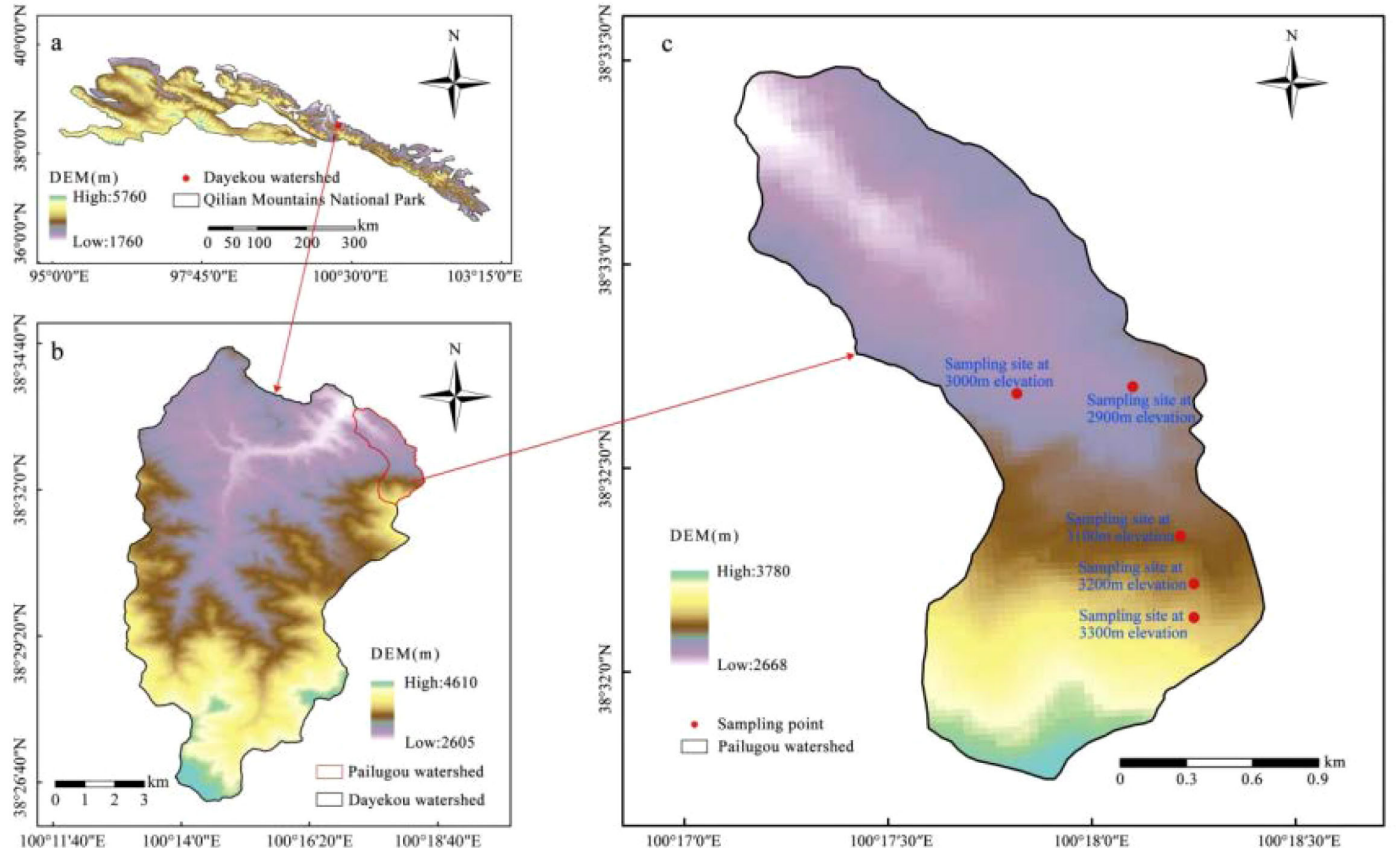
Location of sampling sites in the Pailugou watershed. **(A)** Qilian Mountains National Park. **(B)** Dayekou watershed. **(C)** Pailugou watershed and Sampling site.

The study area is situated within the Pailugou watershed of the Xishui National Nature Reserve, managed by the Gansu Qilian Mountain Forest Ecosystem Research Station, on the mid-northern slope of the Qilian Mountains (**Figure 1**). It spans 100°16′-100°18′ E and 38°31′-38°33′ N, with elevations ranging from 2,600 to 3,800 m a.s.l. The watershed extends 4.25 km in length, exhibits a longitudinal gradient of 1:4.2, and encompasses an area of 2.73 km². The region is characterized by a continental semi-arid alpine climate, with a mean annual temperature of 0.5 °C, long-term mean annual precipitation of 435.5 mm, and mean relative humidity of 60%. The dominant tree species is *Picea crassifolia*, which forms extensive pure coniferous stands. Common shrub species include *Caragana jubata*, *Salix cupularis*, *Potentilla glabra*, and *Potentilla fruticosa*. Dominant herbaceous species include *Carex tristachya*, *Iris lactea*, and *Elymus dahuricus*. Along the elevation gradient, dominant soil types include mountain grey-brown forest soil, mountain chestnut soil, meadow soil, subalpine shrub-meadow soil, and alpine cold desert soil (Wan et al., 2017).

### 2.2 Sample collection and chronology development

In October 2021, five minimally disturbed sites exhibiting optimal conditions for tree growth were selected along an elevation transect at 2,900, 3,000, 3,100, 3,200, and 3,300 m a.s.l. within the study area. At each of the five elevation bands, a single 20 m × 20 m permanent plot was established (**Table 1**). Following established dendrochronological protocols (Fritts and Swetnam, 1989), 15 healthy *Picea crassifolia* trees of dominant or co-dominant crown class were selected per plot to minimize biases associated with competition and microsite heterogeneity. From each tree, two increment cores were extracted at breast height (1.3 m above ground) using a 5.15-mm-diameter Pressler increment borer: one oriented parallel to the slope aspect and the other perpendicular to it, to account for potential radial growth asymmetry. A total of 150 increment cores were collected from 75 trees across the five elevation bands.

**Table 1:** Basic characteristics of sample plots of *Picea crassifolia* forest.

Elevation (m)	Geographic location	Slope (°)	Slope aspect (°)	Diameterat breastheight (cm)	Height (m)	Canopy (m)	Canopy density
2900	100°18’06”E 38°32’42”N	25	330	12.33 ± 1.03	5.67 ± 0.86	2.70 ± 0.28	0.72 ± 0.12
3000	100°17’49”E 38°32’41”N	18	23	15.22 ± 2.19	7.60 ± 1.54	3.15 ± 0.23	0.69 ± 0.09
3100	100°18’13”E 38°32’20”N	18	20	23.67 ± 4.15	6.87 ± 1.05	3.54 ± 0.53	0.60 ± 0.17
3200	100°18’15”E 38°32’13”N	33	15	29.80 ± 5.77	8.87 ± 2.30	4.07 ± 0.64	0.50 ± 0.17
3300	100°18’15”E 38°32’08”N	34	23	4.87 ± 0.95	6.30 ± 1.32	4.68 ± 1.32	0.12 ± 0.03

**Figure 2:**
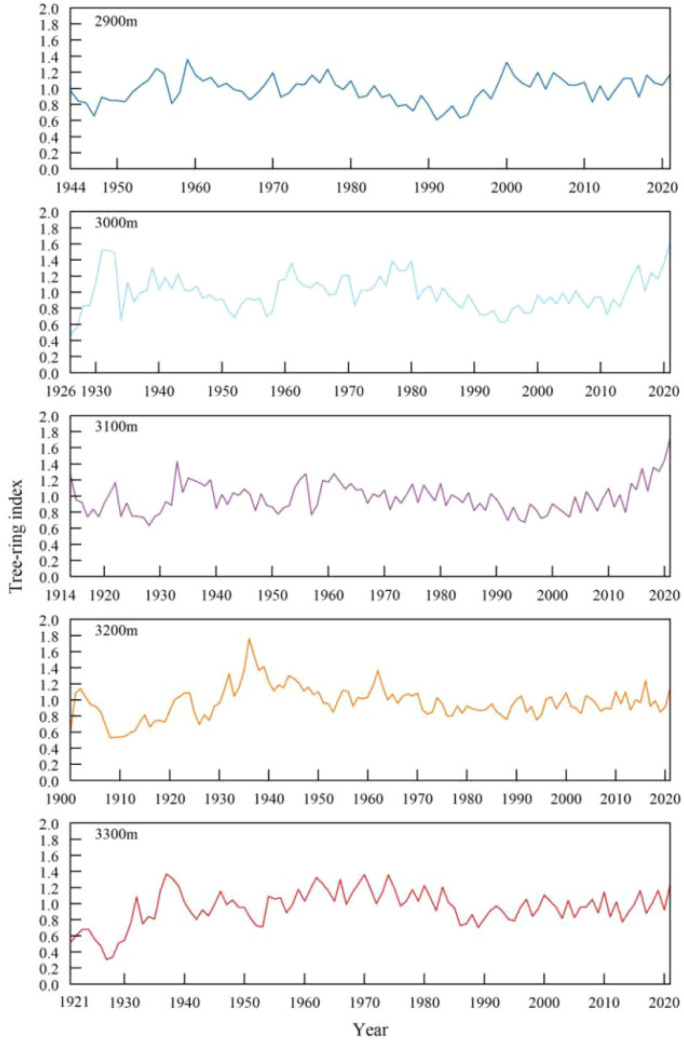
Tree-ring indices of *Picea crassifolia* at the five different elevations of the Pailugou watershed.

All increment cores were transported to the laboratory and processed following established dendrochronological protocols (Wu, 1990). Following air-drying, mounting on wooden supports, and progressive sanding to enhance cellular boundary visibility, tree-ring widths were measured using a LINTAB™ 6 high-precision measuring system (Rinntech, Heidelberg, Germany) with an accuracy of ±0.001 mm. Cross-dating was subsequently performed following Stokes (1996) to verify annual ring assignment. The raw ring-width series were subjected to quality control using the COFECHA program (Holmes, 1983) to verify cross-dating accuracy and identify potential dating errors. Standardized residual chronologies were developed using the ARSTAN software package (Shao and Wu, 1994). Individual ring-width series were detrended using a negative exponential curve or a linear regression of negative slope, as appropriate. Owing to its superior retention of high-frequency climatic signals (Shao and Wu, 1994), the standardized residual chronology type was selected for all subsequent analyses. The resulting tree-ring index series for *Picea crassifolia* across the five elevation bands in the Pailugou watershed are presented in **Figure 2**.

### 2.3 Climate data

Owing to pronounced topographic complexity and substantial elevation mismatches between sampling sites and the nearest instrumental weather stations, we utilized the CRU TS (Climatic Research Unit Time Series) gridded climate dataset. This dataset is robustly validated for capturing regional climate variability in the mountainous regions of northwestern China, offering long temporal coverage, consistent data quality (Wen et al., 2006; Huang et al., 2013; Lyu et al., 2020). Accordingly, this study extracted four monthly climate variables-mean, maximum, and minimum temperature, and total precipitation-for the period 1960–2021 from the CRU TS dataset accessed through the KNMI Climate Explorer (https://climexp.knmi.nl/). The data, provided at a spatial resolution of 0.5° × 0.5°, were spatially averaged over the bounding box 38.52°-39.02°N, 100.27°-100.77°E, which fully encompasses the Pailugou watershed.

Nevertheless, despite its advantages for long-term regional climate analysis, the relatively coarse spatial resolution of CRU data may limit its ability to resolve fine-scale climatic heterogeneity driven by local topography-particularly in areas with complex terrain and large elevation gradients such as our study site. Specifically, microclimatic influences related to slope gradient and aspect may not be adequately captured, potentially affecting the precision of our characterization of elevation-dependent tree growth-climate relationships. To evaluate this potential bias, this study referenced prior assessments of CRU data performance in China. For example, Wen et al. (2022) demonstrated strong concordance between CRU estimates and station observations in the western Tibetan Plateau. Future work will benefit from integrating higher-resolution meteorological datasets to refine and improve the accuracy of growth-climate response models.

**Figure 3:**
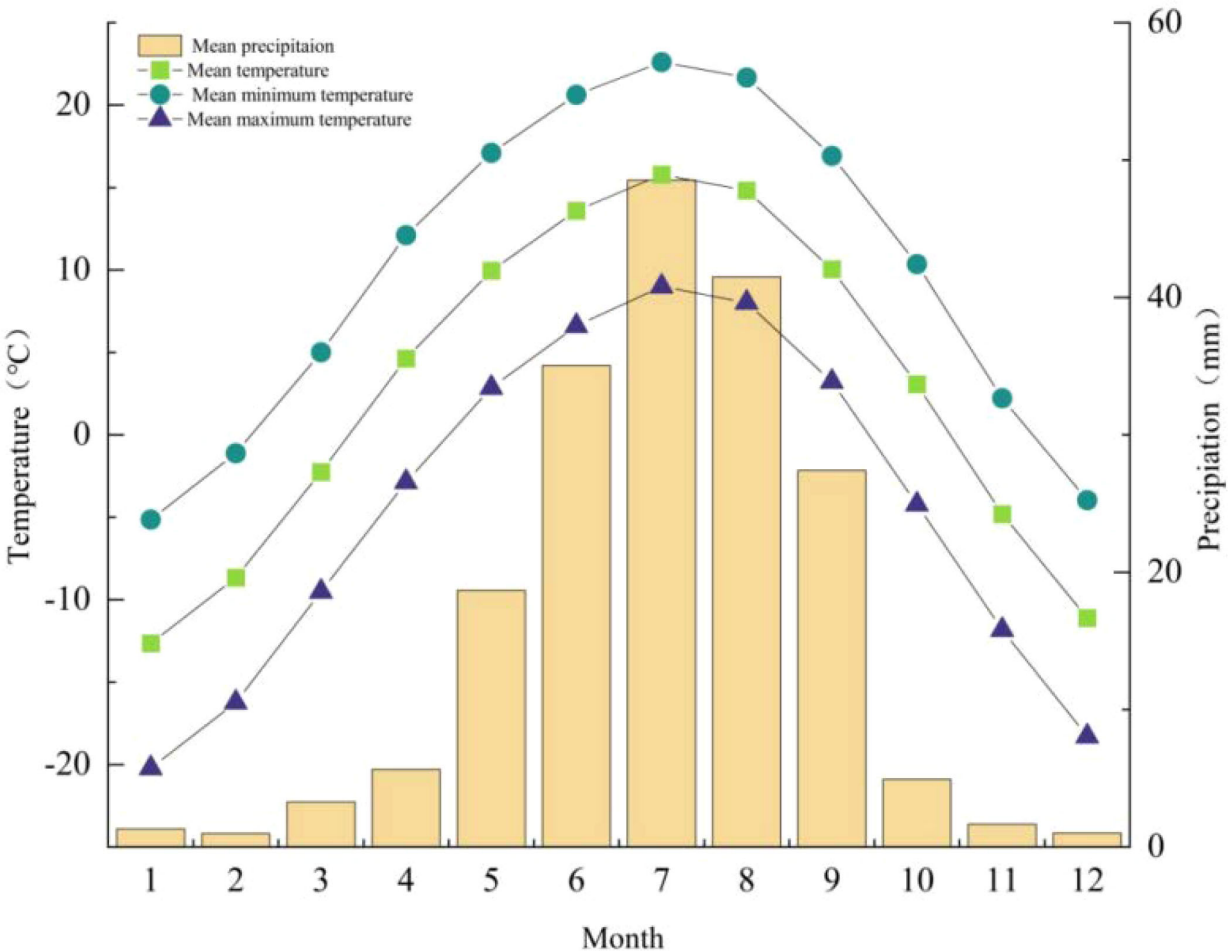
Monthly variations in temperature and precipitation in the Pailugou Watershed of the Qilian Mountains.

**Figure 4:**
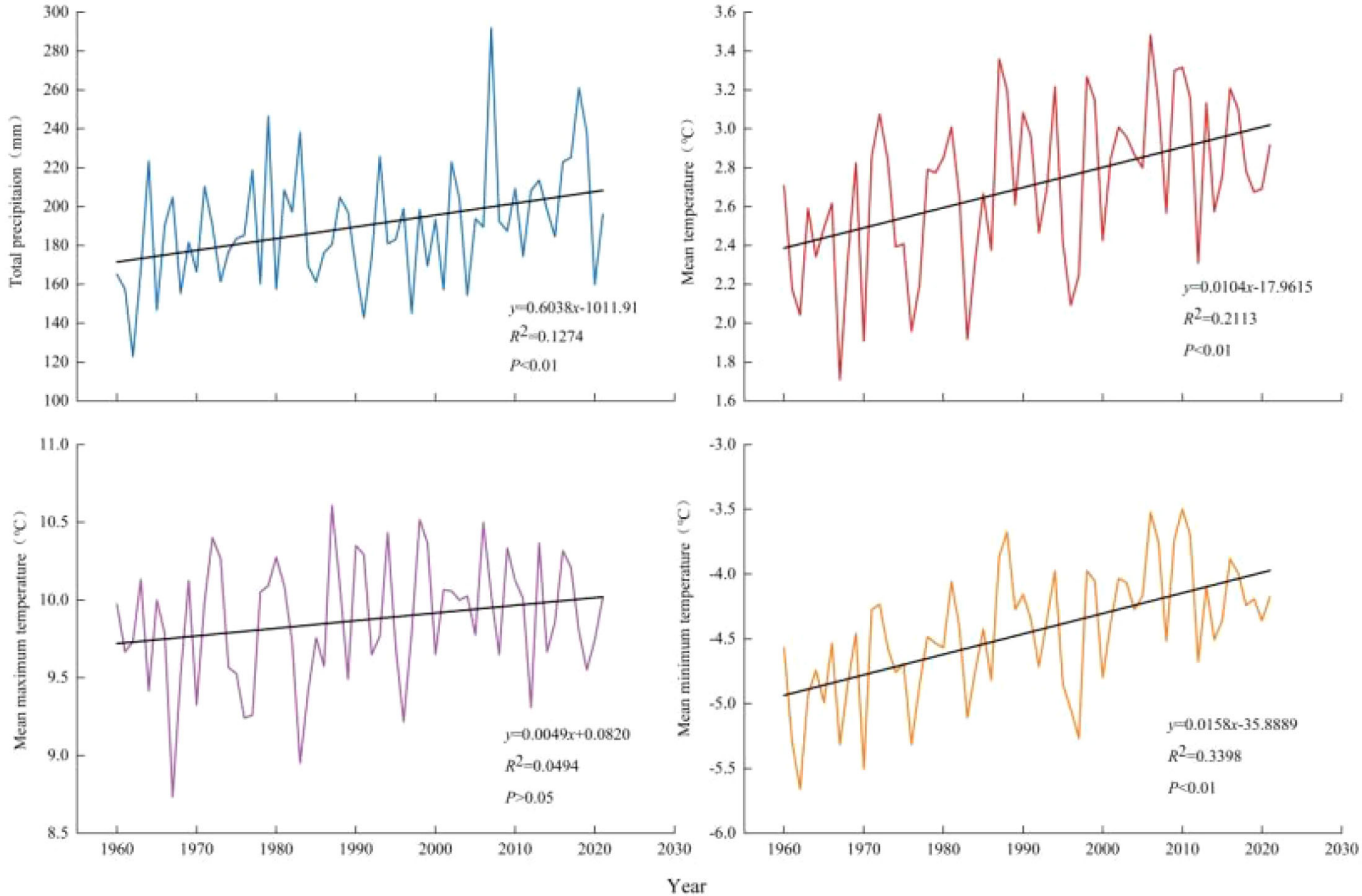
Trends in mean annual temperature and annual total precipitation in the Pailugou Watershed of the Qilian Mountains.

CRU TS data (1960-2021; **Figure 3** and **4**) indicate a mean annual temperature of 2.70°C for the Pailugou watershed, with mean annual maximum and minimum temperatures of 10.61°C and -4.45°C, respectively. July was the warmest month, with mean, mean maximum, and mean minimum temperatures of 15.8 °C, 22.6 °C, and 9.0 °C, respectively. In contrast, January was the coldest month, with mean, mean maximum, and mean minimum temperatures of -12.65 °C, -5.13 °C, and -20.21 °C, respectively. Mean monthly precipitation peaked in July (48.5 mm) and reached a minimum in February (0.99 mm). The long-term mean annual precipitation totaled 189.96 mm, of which 78.6% fell during the five-month period from April to August. From 1960 to 2021, mean annual temperature, mean annual minimum temperature, and annual precipitation all exhibited statistically significant increasing trends (p < 0.01). whereas mean annual maximum temperature displayed a non-significant positive trend (p > 0.05).

### 2.4 Statistical analysis

Chronology reliability was evaluated using standard dendrochronological statistics, specifically the signal-to-noise ratio (SNR) and the expressed population signal (EPS), which quantify the extent to which the sample chronology represents the theoretical population chronology. Higher SNR and EPS values reflect a greater proportion of common variance attributable to shared environmental influences. Following established convention (Wigley et al., 1984), an EPS threshold of ≥0.85 was applied to determine the suitability of each chronology for climate-growth response analyses.

To assess temporal stability and inter-elevation divergence in growth synchrony across the elevation gradient, sliding Pearson correlation coefficients were computed among the five elevation-band chronologies. Concurrently, static Pearson correlations and 30-year sliding-window Pearson correlations (30-year window, 1-year step) were calculated between each chronology and monthly climate variables spanning September of the previous year to October of the current year, to evaluate the strength and temporal stability of climate-growth relationships across elevation bands. Static Pearson correlations were performed using IBM SPSS Statistics (version 27), whereas 30-year sliding-window correlations were conducted with Dendroclim2002 (Biondi and Waikul, 2004), a standard tool in dendroclimatology. All graphical outputs were generated using OriginPro 2024.

## 3 Results

### 3.1 Characteristics of standard chronologies for *Picea crassifolia* at different elevations

Dendrochronological statistics for the five *Picea crassifolia* chronologies across the five elevation bands (**Table 2**) were evaluated to assess their reliability and common signal strength. Chronology robustness over time was assessed using Subsample Signal Strength (SSS), with an SSS threshold of ≥0.85 defining the reliable period for each chronology (Wigley et al., 1984). All five chronologies maintained EPS values > 0.85 throughout their full lengths, confirming their suitability for climate-growth response analyses.

**Table 2:** Statistical characteristics of standard chronologies for *Picea crassifolia* at different elevations.

Statistical features	2900m	3000m	3100m	3200m	3300m
Sequence length	1944-2021	1926-2021	1914-2021	1900-2021	1921-2021
Mean sensitivity(MS)	0.130	0.148	0.149	0.110	0.155
Standard deviation(SD)	0.162	0.222	0.190	0.203	0.218
First-order serial autocorrelation(AC)	0.561	0.552	0.483	0.760	0.680
Correlation coefficient for all series(R1)	0.327	0.419	0.495	0.305	0.465
Correlation coefficient within series(R2)	0.557	0.636	0.587	0.440	0.829
Correlation coefficients between trees(R3)	0.317	0.409	0.484	0.295	0.409
Variance in first eigenvector(PC1)	0.409	0.494	0.582	0.416	0.588
Signal-to-noise ratio(SNR)	6.810	8.652	8.832	4.381	5.210
Expressed population signal(EPS)	0.885	0.896	0.898	0.886	0.860
First year of SSS>0.85(number of tree)	1946(6)	1939(5)	1931(4)	1901(6)	1926(3)

The chronology from 3,300 m displayed the highest mean sensitivity (MS = 0.155), reflecting greater responsiveness of radial growth to interannual environmental fluctuations at this upper treeline location. At 3,200 m, the first-order autocorrelation (AC_1_ = 0.760) was highest among elevations, indicating stronger carry-over effects from prior-year climate on current-year growth. The 3,100 m chronology exhibited the highest interseries correlation $\bar{r}$ = 0.512), signal-to-noise ratio (SNR = 28.6), and expressed population signal (EPS = 0.94), collectively indicating superior common signal strength and rendering it particularly well-suited for detecting regional climate signals.

### 3.2 Relationships between *Picea crassifolia* tree-ring width and climate variables at different elevations

Pearson correlation analysis of the five *Picea crassifolia* chronologies over their shared interval (1960-2021) revealed distinct patterns of growth synchrony along the elevation gradient (**Table 3**). The strongest synchrony occurred between the 2,900 m and 3,000 m elevation bands (r = 0.547, p < 0.001). The 3,100 m chronology exhibited significant positive correlations with the chronologies at 2,900 m (r = 0.386, p < 0.001) and 3,000 m (r = 0.389, p < 0.001), indicating robust regional growth synchrony across the lower and mid-elevation bands. At higher elevations, a significant but weaker positive correlation was observed between the 3,200 m and 3,300 m chronologies (r = 0.224, p < 0.05), consistent with shared climatic constraints near the upper treeline. Conversely, no significant association was detected between the 3,000 m and 3,200 m chronologies (r = −0.006, p > 0.05), suggesting an elevation threshold or ecotone that decouples growth responses between these zones.

**Table 3:** Correlation coefficients between *Picea crassifolia* chronologies at different elevations.

Elevation	2900m	3000m	3100m	3200m	3300m
2900m	1				
3000m	0.547^**^	1			
3100m	0.386^**^	0.389^**^	1		
3200m	0.066	-0.006	0.037	1	
3300m	-0.037	0.045	0.114	0.224^*^	1

* *p* < 0.05; ** *p* < 0.01.

**Figure 5:**
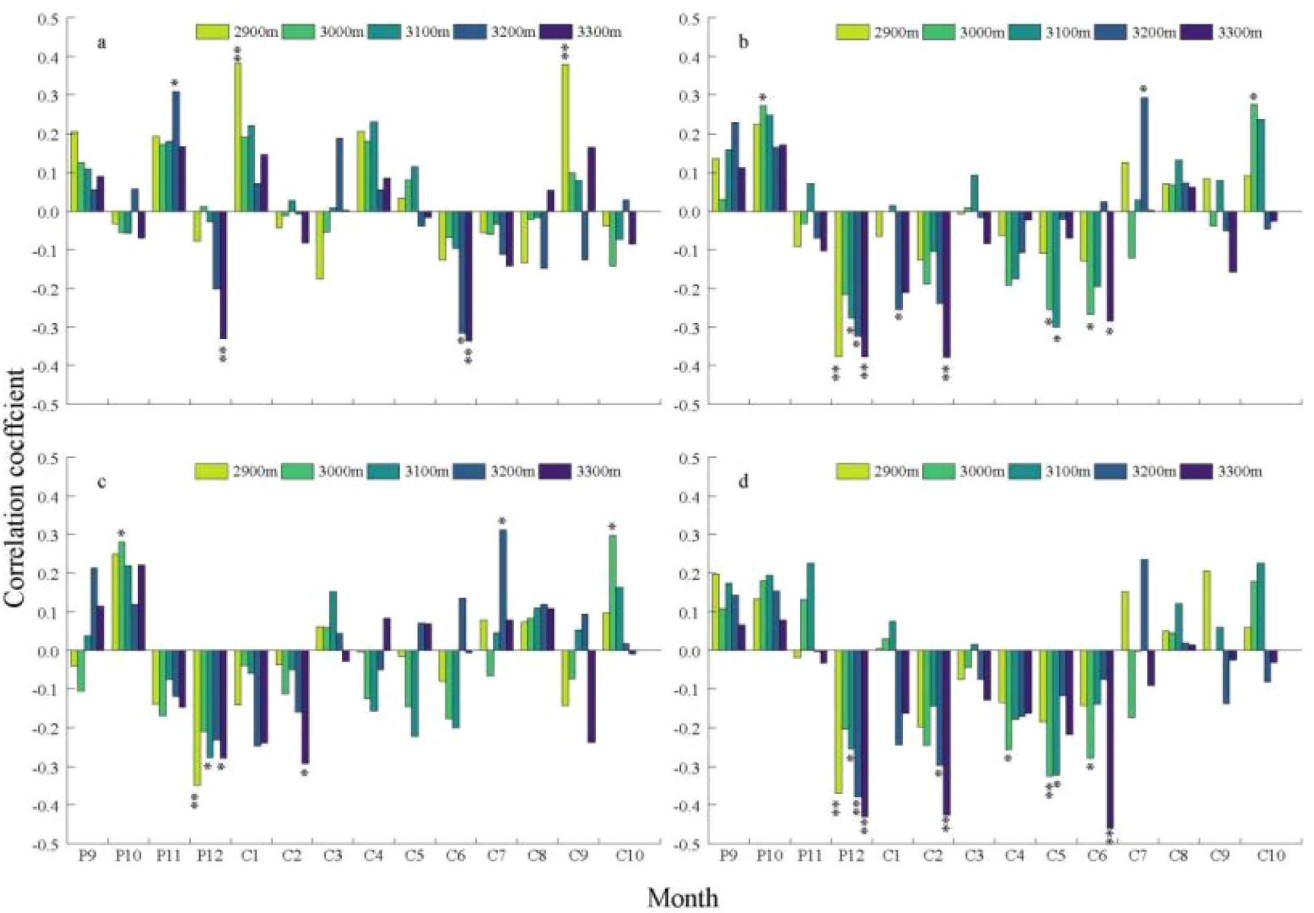
Correlations between *Picea crassifolia* chronologies at different elevations and monthly climatic factors. **(A)** Precipitation. **(B)** Mean temperature. **(C)** Mean maximum temperature. **(D)** Mean minimum temperature. P, Previous year; C, Current year. (*p < 0.05; **p < 0.01).

Growth-climate analyses revealed that radial growth of *Picea crassifolia* across the Pailugou watershed is co-limited by temperature and precipitation, with climate sensitivity exhibiting pronounced shifts along the elevation gradient (**Figure 5**). At the upper treeline (3,300 m elevation band), radial growth exhibited negative associations with warm and wet conditions during the cold season and early summer. Significant negative correlations (p < 0.01) with prior-year December and current-year June precipitation, and with December and February temperatures, suggest that elevated winter warmth and excess early-summer moisture may suppress growth-potentially through increased respiratory carbon losses or reduced snowpack insulation. At 3,200 m, radial growth displayed a bimodal response pattern: positive correlations (p < 0.05) with prior-year November precipitation and July temperatures were offset by negative correlations (p < 0.05) with June precipitation and December temperatures, reflecting competing influences of autumn moisture recharge, growing-season warmth, and winter thermal dynamics. At 3,100 m, radial growth showed consistent negative associations with winter and spring temperatures (significant for all December temperature variables and May; p < 0.05), implying that early-season warmth may advance snowmelt or elevate evaporative demand. At 3,000 m, growth exhibited a positive relationship with October temperatures (p < 0.05), underscoring autumn’s role in carbohydrate storage and cambial priming; conversely, negative correlations with May and June temperatures (p < 0.05), particularly May minimum temperature (p < 0.01), indicate vulnerability to cold extremes during early summer. At the lowest elevation band (2,900 m), radial growth was significantly associated with non-growing-season moisture and winter thermal conditions, showing strong positive correlations with January and September precipitation (p < 0.01) and negative correlations with December temperatures (p < 0.01), suggesting winter warming may intensify moisture stress via enhanced snow sublimation or premature melt.

These growth-climate relationships derive from CRU TS gridded data (0.5° × 0.5° resolution). Although validated for regional climate patterns in northwestern China, this resolution may not resolve microclimatic heterogeneity driven by local topography (e.g., slope, aspect, elevation-induced thermal inversions) among sampling sites. Consequently, fine-scale variation in actual climate exposure across elevation bands may be underestimated, potentially influencing the precision of inferred elevation-dependent response patterns.

### 3.3 Temporal stability of climate-growth relationships across elevation bands

Thirty-year sliding-window correlation analyses were conducted for each elevation band (2,900–3,300 m) over the common interval of 1960–2021 to assess the temporal stability of climate-growth relationships. Results revealed pervasive non-stationarity in radial growth responses to climate, with the magnitude and seasonal expression of non-stationarity varying systematically across the elevation gradient.

**Figure 6:**
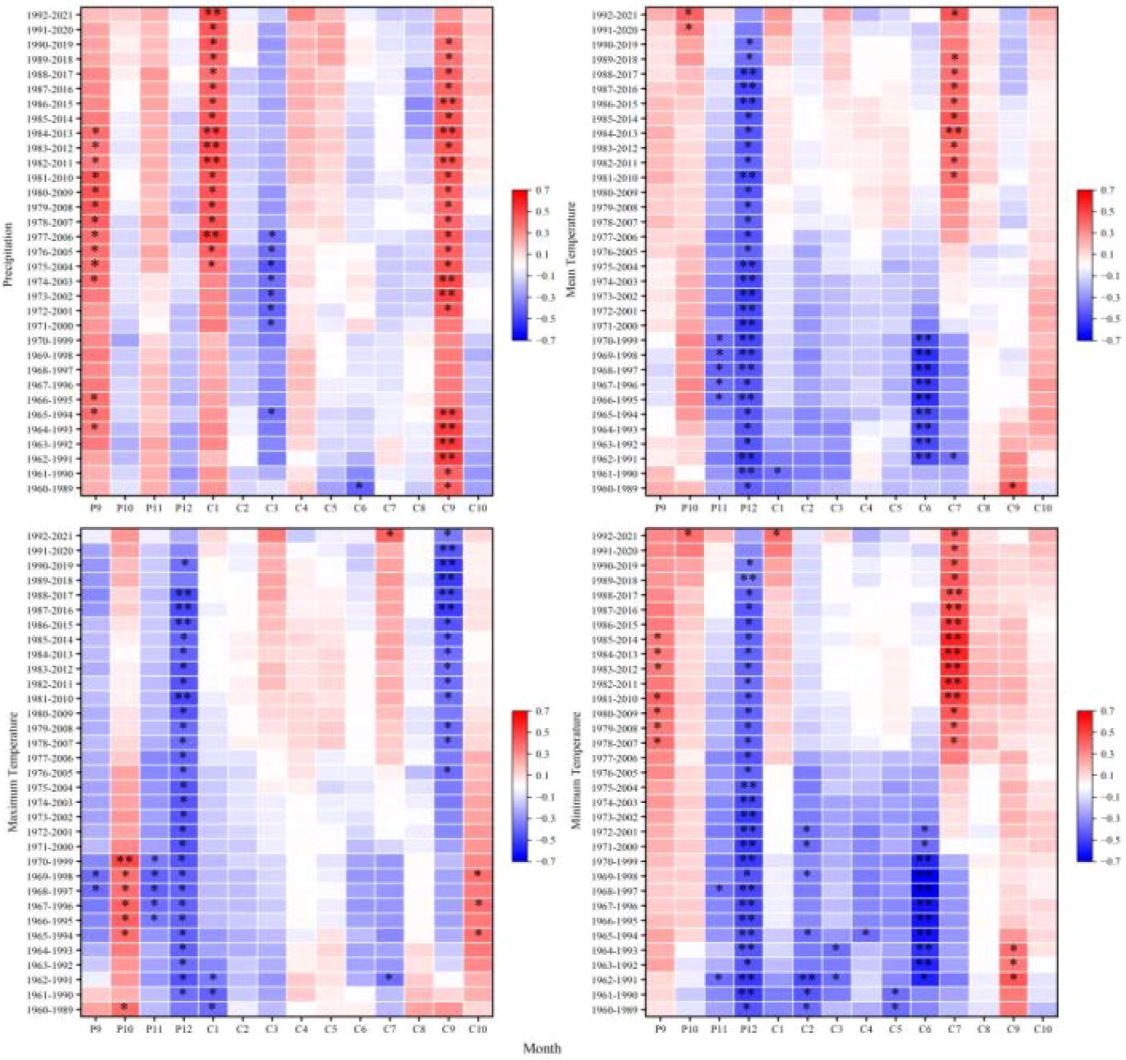
Moving correlations between the standardized chronology of tree-ring width for *Picea crassifolia* at 2900 m elevation and precipitation, mean temperature, maximum temperature, and minimum temperature.

At the 2,900 m elevation band, climate-growth correlations exhibited significant temporal non-stationarity, particularly for previous-year September and current-year January and March precipitation; June-July temperatures; and previous-year October-December thermal conditions. Even correlations previously considered stable (e.g., with September precipitation) became non-stationary, suggesting that recent environmental changes may be decoupling tree growth from historical climate drivers (**Figure 6**).

**Figure 7:**
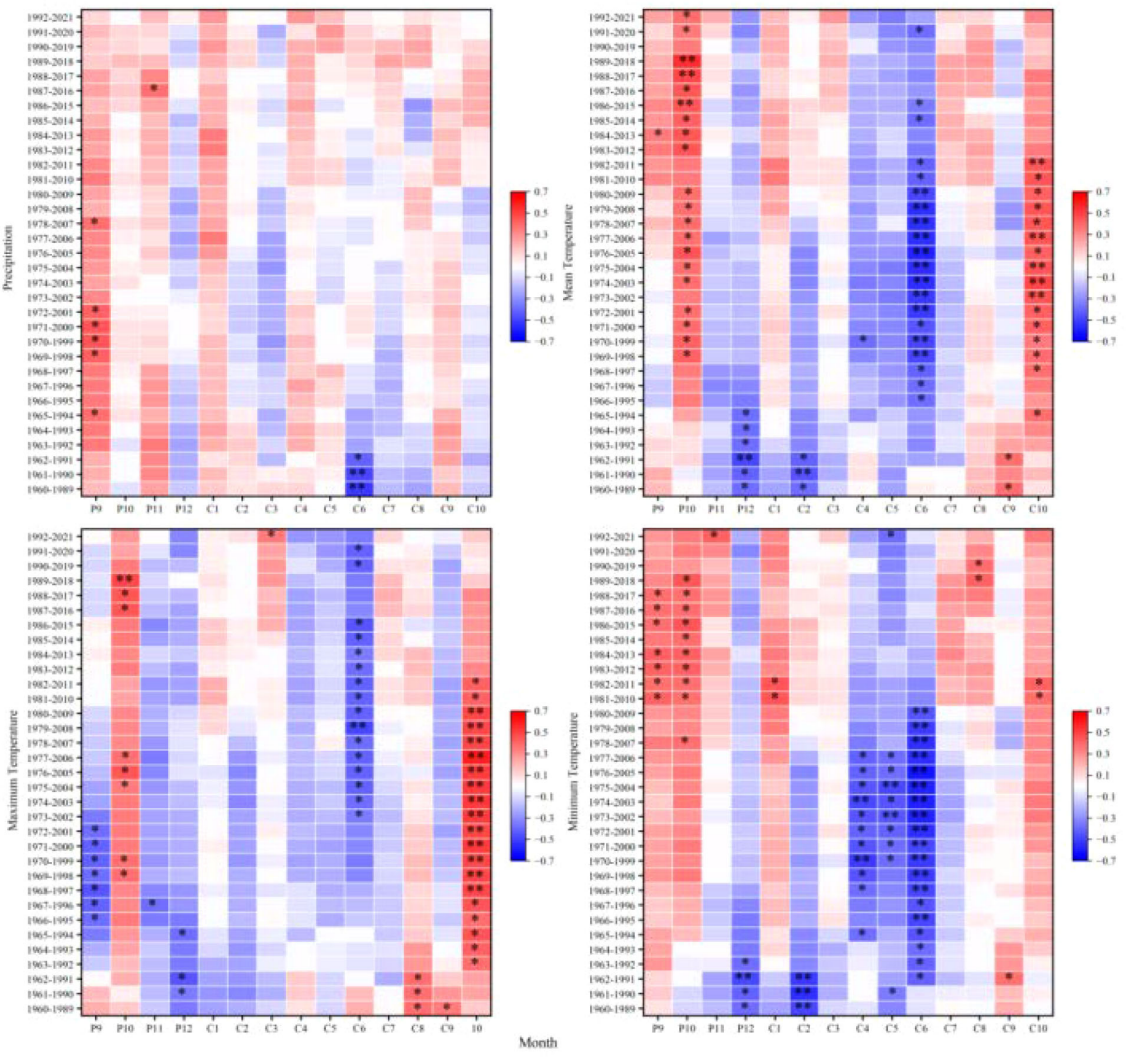
Moving correlations between the standardized chronology of tree-ring width for *Picea crassifolia* at 3000 m elevation and precipitation, mean temperature, maximum temperature, and minimum temperature.

At 3,000 m, climate-growth relationships exhibited marked non-stationarity. Precipitation correlations, particularly for previous-year September and current-year March, exhibited significant non-stationarity. Similarly, temperature correlations showed pronounced non-stationarity, particularly for previous-year September-December and current-year March, June, and October temperatures. Responses to minimum temperature also exhibited non-stationarity, suggesting potential reorganization of frost-risk phenology or spring warming effects at this transitional elevation band (**Figure 7**).

**Figure 8:**
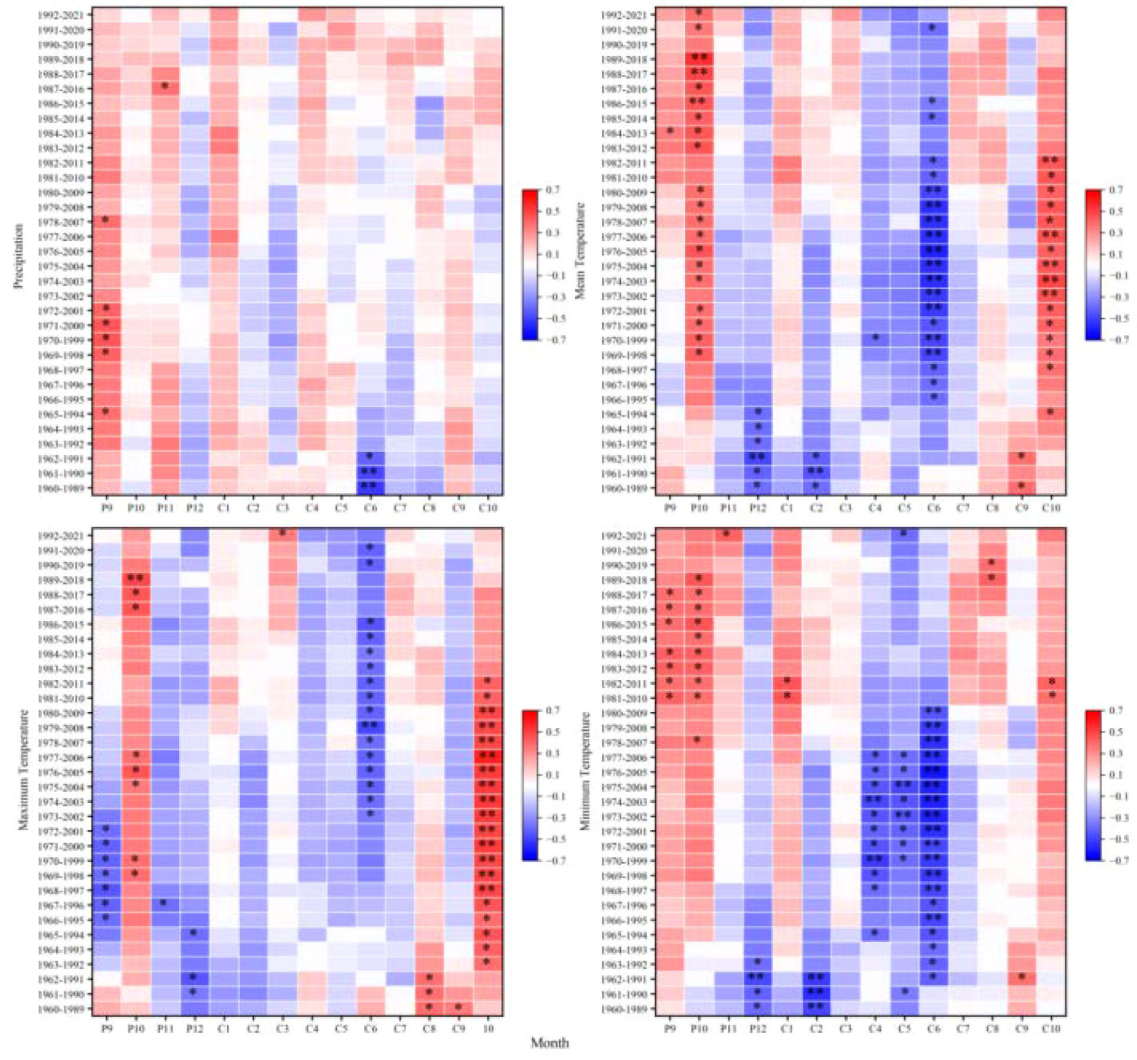
Moving correlations between the standardized chronology of tree-ring width for *Picea crassifolia* at 3100 m elevation and precipitation, mean temperature, maximum temperature, and minimum temperature.

At 3,100 m-previously identified as exhibiting the strongest common climate signal-climate-growth relationships nonetheless displayed pronounced non-stationarity. Precipitation correlations varied during critical moisture-recharge (September) and growing-season (June) periods, while temperature sensitivities fluctuated markedly for previous-year September-December and current-year June, August, and October. Inconsistent responses to minimum temperature further indicate disruption of previously coherent growth patterns at this mid-elevation band (**Figure 8**).

**Figure 9:**
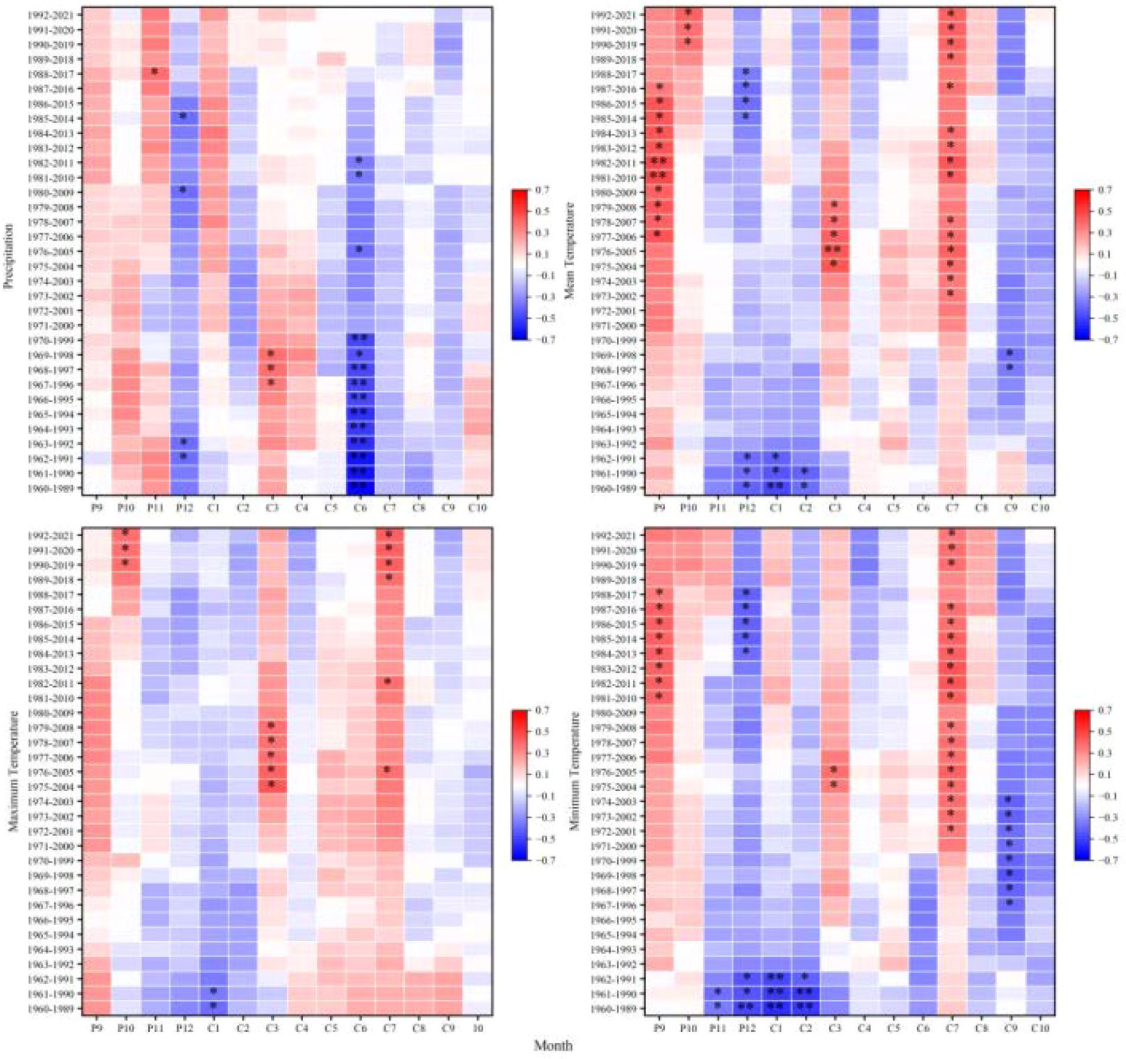
Moving correlations between the standardized chronology of tree-ring width for *Picea crassifolia* at 3200 m elevation and precipitation, mean temperature, maximum temperature, and minimum temperature.

At 3,200 m, substantial non-stationarity affected both moisture and thermal responses. Precipitation correlations shifted for previous-year December and current-year March-June, reflecting altered snowpack dynamics and early-growing-season moisture availability. Temperature sensitivities also varied notably during the previous-year September-December period and for current-year July, suggesting increasing counteraction between warm summers and drought stress or phenological shifts (**Figure 9**).

**Figure 10:**
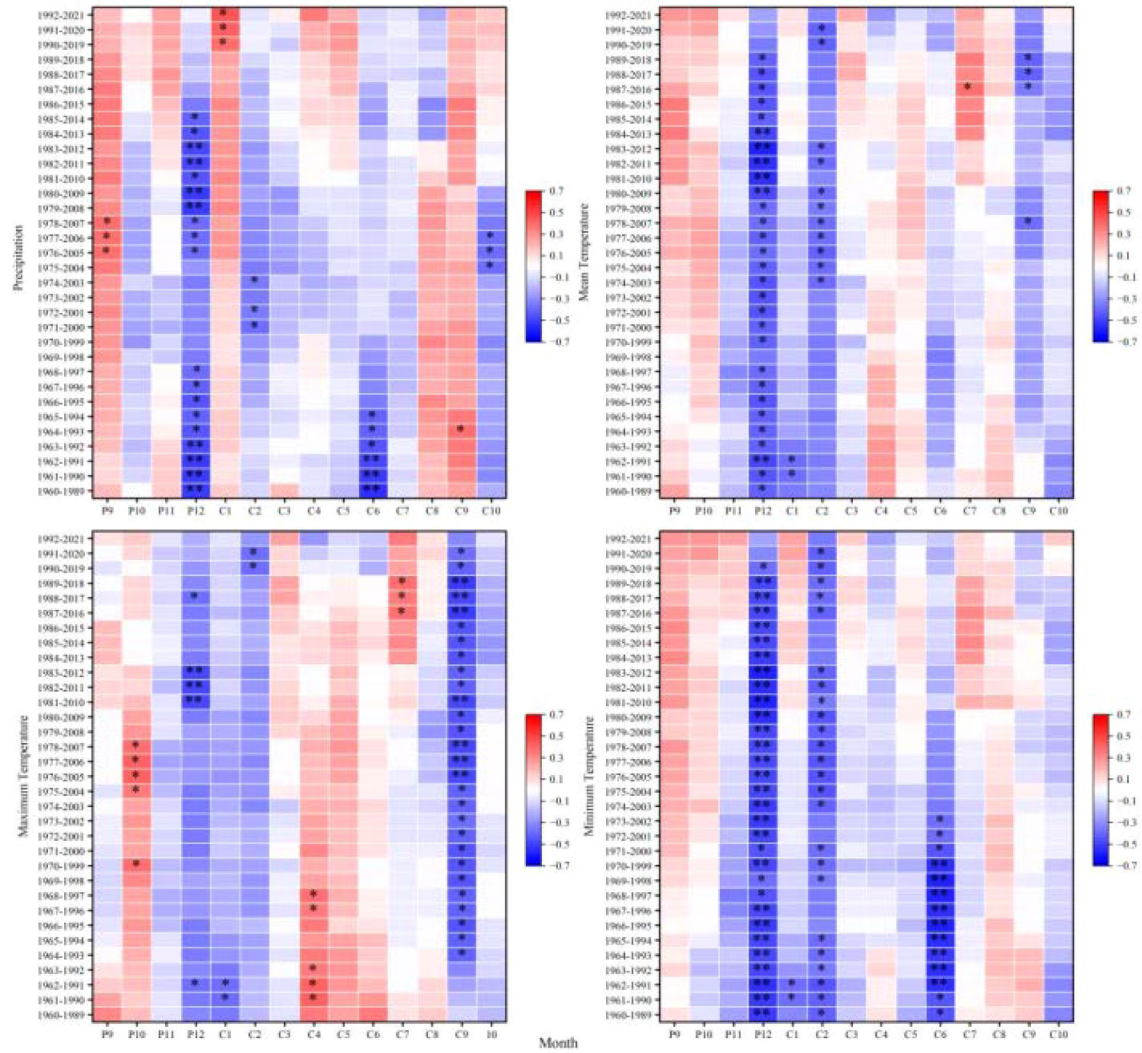
Moving correlations between the standardized chronology of tree-ring width for *Picea crassifolia* at 3300 m elevation and precipitation, mean temperature, maximum temperature, and minimum temperature.

At the upper treeline (3,300 m elevation band), non-stationarity was comparatively constrained, affecting fewer variables and primarily marginal seasonal windows. Precipitation correlations fluctuated for previous-year September-December and current-year January-February, June, and October, likely reflecting variable snowpack persistence. Temperature-related non-stationarity was largely confined to December and February-June, indicating evolving influences of winter cold and early-summer thermal conditions; nevertheless, the role of temperature as a limiting factor near the treeline exhibited greater temporal stability relative to lower elevation bands (**Figure 10**).

## 4 Discussion

This study presents a comprehensive analysis of *Picea crassifolia* radial growth responses to climate change across an elevational gradient in the Qilian Mountains, with particular emphasis on the influences of temperature and precipitation variability on tree growth. The results demonstrate that radial growth-climate relationships vary significantly with elevation and exhibit pronounced temporal non-stationarity. These findings provide critical insights into how climate change influences forest ecosystem dynamics along elevational gradients.

### 4.1 Influence of elevational gradient on *Picea crassifolia* growth

Elevation, a critical ecological driver of tree growth, governs the redistribution of heat and moisture, thereby increasing environmental complexity (Cao et al., 2014). Conventional understanding posits that tree growth at lower elevations is primarily moisture-limited (exhibiting positive correlations with precipitation), whereas growth at higher elevations is typically temperature-limited (showing positive responses to warming) (Wu, 1990). Contrary to this classical paradigm, radial growth across the elevational gradient exhibited consistent negative correlations with temperatures during the previous-year December, current-year January-February, and May-June. This pattern aligns with findings by Liu et al. (2021) in the eastern Qilian Mountains, where *Picea crassifolia* radial growth at mid- and high-elevations showed significant negative correlations with mean and maximum temperatures during the previous-year June-September and current-year February-March and June-August.

At high elevations, low temperatures can significantly impede tree growth not only by restricting photosynthesis but also by disrupting vital physiological processes, including water and nutrient uptake, soil freezing, and carbon allocation (Xue et al., 2023; Rodriguez-Caton et al., 2021). These constraints are further exacerbated by shortened growing seasons and low soil temperatures, which suppress root activity and nutrient uptake (Hu et al., 2024). Furthermore, freeze-thaw cycles induced by low temperatures cause physiological stress through cellular membrane damage and disruption of nutrient transport, thereby further constraining growth (Xue et al., 2023). Consistent with these mechanisms, research on conifer species such as *Picea schrenkiana* at the upper treeline demonstrates that thermal stress-particularly from extreme winter cold-directly compromises tree vitality by impairing root function and nutrient cycling (Liu et al., 2024).

Beyond direct thermal constraints, trees at high elevations exhibit pronounced susceptibility to moisture stress (Sohar et al., 2024). At elevations ≥3,200 m, elevated summer temperatures intensify soil moisture evaporation, thereby reducing water availability critical for sustaining physiological processes including transpiration and photosynthesis (Tonello et al., 2021; Pernicová et al., 2024). Additionally, intense solar radiation prevalent at high elevations-particularly during June-July-exacerbates transpirational water loss, precipitating water deficits that constrain growth (Pernicová et al., 2024). This phenomenon is particularly acute in the Qilian Mountains, where limited precipitation and high evaporative demand collectively impose stringent growing constraints on trees near the upper treeline (Zhang et al., 2023; Niu et al., 2024).

The observed negative temperature-growth correlations at higher elevations contrast with the classical paradigm positing that tree growth at upper elevations is positively temperature-limited (Zhang et al., 2015). Contemporary research underscores the complexity of growth-climate relationships in high-elevation environments, indicating that temperature alone is insufficient to explain growth patterns at these sites (Yin et al., 2023; Altman et al., 2020). Instead, moisture availability-mediated by snowpack dynamics and precipitation seasonality-plays a pivotal role in modulating growth in cold environments (Alonso-González et al., 2020). For example, research on the Tibetan Plateau indicates that while rising temperatures may extend the growing season, they concurrently exacerbate water stress in the absence of sufficient precipitation, ultimately suppressing growth despite thermal amelioration (Pu et al., 2021; Cao et al., 2023). Tree rings, which archive annual growth increments, are highly sensitive to these seasonal fluctuations in temperature and moisture (Xue et al., 2023). The distinct anatomical bands within tree rings provide a detailed chronicle of a tree’s physiological responses to environmental conditions.

At mid-elevations (approximately 3,100 m), tree growth exhibits a more balanced relationship with climatic factors, wherein temperature and moisture interact synergistically to optimize growth (Peng et al., 2022). In these zones, seasonal variations in temperature and moisture directly regulate key physiological processes-including cambial activity and carbohydrate storage-thereby enhancing growth performance (Matula et al., 2023; Soubeyrand et al., 2024). This mid-elevation growth optimum aligns with ecological optimality theory, wherein an optimal balance of energy and water availability fosters peak biodiversity and ecosystem productivity. Cambial activity, which determines annual ring width, represents a direct physiological response to these environmental cues.

Collectively, these findings indicate that at high elevations, the negative growth-temperature correlations are predominantly attributable to indirect mechanisms-such as moisture stress and freeze-thaw dynamics-rather than direct thermal limitations (Xu et al., 2024). The interplay of these complex factors necessitates a nuanced, integrated understanding of high-elevation tree responses to climatic variability, particularly under persistent global warming (Schnabel et al., 2021).

### 4.2 Differences in the response of *Picea crassifolia* radial growth to climate across elevations

This study reveals pronounced temporal non-stationarity in climate-growth relationships, a pattern most evident across mid- to high-elevation sites. The magnitude and direction of correlations between tree growth and climatic variables display substantial seasonal variation, mediated predominantly by the interactive effects of temperature and precipitation. Such non-stationarity is accentuated at higher elevations due to distinct climatic regimes and physiological constraints inherent to these environments (He et al., 2024; Altman et al., 2020).

The temporal non-stationarity documented in the Qilian Mountains is not an isolated phenomenon (Wei et al., 2023; Qin et al., 2024). Comparable patterns have been reported across diverse high-elevation ecosystems. For instance, research on *Picea schrenkiana* tree-ring chronologies in the Alatau Mountains revealed significant temporal shifts in climate-growth associations, with pronounced non-stationarity during critical phenological windows of the growing season. Similarly, studies on the northern Tibetan Plateau demonstrated that *Larix gmelinii* growth exhibits high sensitivity to temperature and moisture fluctuations, with climate-growth linkages dynamically reconfiguring under regional warming trends (Du et al., 2024).

A primary mechanism underlying this non-stationarity involves the complex interplay between temperature and moisture availability, particularly evident in the seasonal progression of climatic variables. In high-elevation settings characterized by pronounced thermal variability, key phenological events-including snowmelt timing, growing season initiation, and persistence of suboptimal thermal conditions in late spring and early summer-substantially influence growth processes (Sohar et al., 2024). Empirical evidence from alpine conifer studies in the European Alps and Rocky Mountains indicates that late-spring frost events, often linked to climate warming, disrupt cambial activity and destabilize growth trajectories. Furthermore, temperature-mediated shifts in snowmelt phenology alter the temporal distribution and availability of soil moisture-a resource pivotal for early-season growth-thereby amplifying climate-growth response variability (Wang et al., 2024).

Temporal non-stationarity is especially pronounced at mid-elevations (3,100 m), an ecotonal zone where temperature-mediated drought stress and moisture availability intersect. This convergence generates marked fluctuations in growth responses (Wang et al., 2019). Analogous patterns have been documented in central Tianshan Mountain tree-ring studies, wherein mid-elevation trees displayed highly variable growth sensitivities to seasonal climatic fluctuations. Such variability likely reflects dynamic shifts in limiting factors: under favorable conditions, synergistic temperature-moisture interactions promote growth; under adverse conditions, their combined effects impose physiological constraints (Liang et al., 2021; Wang et al., 2021).

At the uppermost elevational range (3,200-3,300 m), non-stationarity in growth responses to winter cold and spring warming is consistent with findings from other cold-temperate and alpine regions (Zhang et al., 2015, 2017). Swiss Alps research demonstrates that high-elevation trees exhibit complex growth patterns driven by synergistic effects of low temperatures and snowpack dynamics, yielding highly variable microclimatic conditions. Concurrently, Rocky Mountain studies establish that elevated winter precipitation coupled with warmer spring temperatures intensifies freeze-thaw cycles, impairing critical physiological processes (Kellerer-Pirklbauer, 2017). Collectively, these findings indicate that growth responses at high elevations are governed not solely by mean climatic conditions but also by the frequency and intensity of extreme seasonal events-such as late-spring frosts or premature snowmelt-which introduce additional complexity into climate-growth relationships (Kellerer-Pirklbauer, 2017).

The mechanisms driving these non-stationary responses are inherently multifaceted. Primary determinants encompass alterations in water availability (mediated by snowmelt phenology and precipitation regimes) and temperature-induced physiological constraints (including freeze-thaw dynamics and early-season heat stress) (Xue et al., 2023; Basnet et al., 2024; Zhang et al., 2023). Empirical studies confirm heightened vulnerability of high-elevation trees to spring frost events, which can damage nascent tissues and disrupt seasonal carbon allocation. Moreover, the intricate interplay among soil temperature, moisture dynamics, and root physiological function further complicates growth responses in alpine species. These mechanistic insights align with Qilian Mountain observations, where temporal shifts in the magnitude and timing of temperature and precipitation events have precipitated heightened growth non-stationarity across mid- and high-elevation gradients (Wu et al., 2021).

Dendroclimatology utilizes tree-ring records to reconstruct past climate conditions, with ring width and density serving as quantitative proxies for historical environmental variability. Wide rings typically reflect years with favorable growing conditions-adequate moisture, sufficient solar radiation, and optimal temperatures-whereas narrow rings often indicate climatic or biotic stressors such as drought, cold extremes, or insect outbreaks. Through cross-dating, annually resolved chronologies are constructed while mitigating errors associated with missing or false rings, thereby yielding robust datasets for climate reconstruction. This approach is particularly critical for deciphering tree growth responses to climate change in climatically sensitive high-elevation ecosystems (Zhang et al., 2023).

However, inferences regarding temporal non-stationarity in this study, derived from CRU TS gridded climate data (0.5° × 0.5° resolution), are subject to notable limitations. Although CRU data exhibit strong long-term consistency at regional scales, their coarse spatial resolution may fail to resolve localized microclimatic dynamics within topographically complex terrain such as the Pailugou watershed. Consequently, portions of the observed non-stationarity may arise from unresolved biases or smoothing artifacts inherent to the climate input data, rather than solely representing genuine shifts in tree physiological or ecological responses. Readers are therefore cautioned against overinterpreting short-term fluctuations, and future investigations are encouraged to integrate higher-resolution observational networks or dynamically downscaled climate products to rigorously validate temporal stability patterns.

## 5 Conclusion

This study demonstrates that climate-growth relationships in *Picea crassifolia* across the Qilian Mountains are highly dynamic and non-stationary, exhibiting significant variation along the elevational gradient. At lower elevations (2,900-3,000 m), tree growth is primarily moisture-limited, with positive correlations to precipitation during the non-growing season. At higher elevations (3,200–3,300 m), growth is more constrained by temperature, with negative correlations observed with winter precipitation and summer temperatures, reflecting sensitivity to thermal constraints and moisture stress. Temporal non-stationarity in growth responses was particularly pronounced at mid- to high-elevations (3,100–3,300 m), where interactions between temperature and moisture stress became increasingly unpredictable under warming trends. These findings underscore the increasing complexity of growth-climate interactions in alpine forests, indicating that both temperature and moisture availability-including their seasonal dynamics-are critical determinants of tree growth. This study highlights the necessity for adaptive forest management frameworks that explicitly incorporate both short-term climatic extremes and long-term shifts in dominant climate drivers.

## Data Availability

The original contributions presented in the study are included in the article/supplementary material. Further inquiries can be directed to the corresponding author.
